# Factors associated with daily walking of dogs

**DOI:** 10.1186/s12917-015-0434-5

**Published:** 2015-05-19

**Authors:** Carri Westgarth, Hayley E. Christian, Robert M. Christley

**Affiliations:** Department of Epidemiology and Population Health, Institute of Infection and Global Health, and School of Veterinary Science, University of Liverpool, Leahurst Campus, Chester High Road, Neston, Cheshire CH64 7TE UK; Centre for the Built Environment and Health, School of Population Health, and Telethon Kids Institute, The University of Western Australia (M707), 35 Stirling Highway, Crawley, WA 6009 Australia; NIHR Health Protection Research Unit in Emerging and Zoonotic Infections, Liverpool, L69 7BE UK

**Keywords:** Dogs, Walking, Physical activity, Cross-sectional study

## Abstract

**Background:**

Regular physical activity is beneficial to the health of both people and animals. The role of regular exercise undertaken together, such as dog walking, is a public health interest of mutual benefit. Exploration of barriers and incentives to regular dog walking by owners is now required so that effective interventions to promote it can be designed. This study explored a well-characterised cross-sectional dataset of 276 dogs and owners from Cheshire, UK, for evidence of factors associated with the dog being walked once or more per day.

**Results:**

Factors independently associated with daily walking included: number of dogs owned (multiple (vs. single) dogs negatively associated); size (medium and possibly large dogs (vs. small) positively associated); and number of people in the household (more people negatively associated). Furthermore, a number of factors related to the dog-owner relationship and the dog’s behaviour were associated with daily walking, including: having acquired the dog for a hobby (positively associated); dog lying on furniture (positively associated); dog lying on laps (negatively associated); growling at household members (negatively associated); and playing chase games with the dog (negatively associated).

**Conclusions:**

These findings are consistent with the hypothesis that the strength and nature of the human-dog relationship incentivises dog walking, and that behavioural and demographic factors may affect dog walking via this mechanism. Future studies need to investigate how dog demographic and behavioural factors, plus owner behavioural factors and perceptions of the dog, influence the dog-human relationship in respect to the perceived support and motivation a dog can provide for walking.

## Background

Regular physical activity is beneficial for the health of both people and animals, and has a role in preventing and treating numerous causes of morbidity and mortality [1]. With rising levels of obesity in both humans [2] and dogs [3], the potential of regular exercise undertaken together, such as dog walking, has come to the fore as a realistic public health intervention that benefits both humans and dogs [4].

Numerous studies have confirmed that dog owners are more physically active than those without dogs, but also that not all pet dogs are walked regularly (for a review of this evidence, see [4]). More recently attention has turned to exploring the barriers and incentives to regular dog walking using both qualitative and quantitative research methods [5] in order to identify modifiable factors that can be used in interventions to encourage dog walking [6–9]. Most research to date has been conducted in North America (e.g. [10–17]) or Australia (e.g. [18–23] with relatively limited data from the UK [9, 24, 25]. However, cultural differences regarding dog owning and walking practices means that findings from one country do not necessarily apply to another. For example in some countries such as the US fenced ‘dog parks’ are commonly used as off leash exercise areas [26] whereas in the UK and Australia these are rare.

A recent review of the correlates of dog walking highlights that one of the most important influences on how often an owner walks their dog is the strength of the relationship the owner has with the dog [5]; this is often referred to as ‘a sense of obligation’ to walk the dog [10, 14] or reporting that the dog provides support and motivation for walking [21, 27]. Evidence of the influence of dog-related factors such as number of dogs and size, was mixed, but may be due to the already accounted for influence of the level of motivation a dog provides for walking [5]. Physical environment factors may also play an important role in encouraging physical activity in people in general, but also through the provision of walking areas with specific dog-supportive features [5, 21, 27]. Another area that requires further investigation is the role of the facilitation of social interactions that dog walking can provide [5, 28, 29].

The majority of studies in this area have focused on the owner as the activity subject of interest, rather than the dog [5]. However a disadvantage to this approach has been that the datasets providing the information are scarce on dog-specific and owner-dog relationship information. Datasets collected with the dog as the focus instead have the potential to provide more in-depth detail surrounding the influence of dog demographic and behavioural factors, dog management factors, and owner beliefs about the dog. The objective of this study was to describe dog walking and explore the factors associated with it, in particular the type and strength of owner-dog relationship, using a well- characterised UK dataset of dogs and their owners [24, 25, 30, 31].

## Methods

Ethical approval for the analysis was obtained from the Liverpool Veterinary School Ethics Committee (VREC-75, 13/12/2012). Owners consented to providing the information by completing the questionnaire after reading an information sheet.

### Data collection

Data collection has been described extensively previously [24, 30]. Briefly, doorstep interviews with 1278 households in a defined geographic community in Cheshire, UK, identified 260 dog owning households that were subsequently recruited to self-complete a questionnaire survey about owner and dog behaviour and general management. Data was collected in the period July – October 2005. Much detail was collected on dog walking practices and on dog and owner behaviours that may contribute to, or be proxies for, the strength of the dog-owner relationship. The dataset comprised of 279 dogs owned by 214 households who participated in the study by returning their questionnaires.

### Data analysis

Data analysis was performed in Minitab® Statistical Software Version 16.0 and IBM SPSS Statistics for Windows Version 21.0. Data were analysed at the level of the dog, for the binary outcome of walking frequency - dog is walked at least once or more per day – referred to as ‘daily dog walking’. Simple univariable associations were examined using chi-squared tests and binary logistic regression for dog demographic, household demographic, management, reasons for getting a dog, dog behaviour and walking behaviour variables (for more description see Tables 1, 2, 3, 4, 5 and 6 and [24, 30]). Variables were selected due to their potential to act as barriers or incentives with dog walking frequency, for example measuring an aspect of the owner-dog relationship or a commitment to animal care. Previously created demographic variables describing the household age structure and occupation type structure were also used [30]. For variables where a cell contained zero because all dogs were walked daily, one dog was randomly selected and changed to walked less than once daily, and univariable analysis performed again.

**Table 1:** Univariable analysis of dog demographic factors associated with daily dog walking

Variable		<1/day	1+/day	OR	95 % CI	P
		n (%)	n (%)			
**Dog type**	Unknown crossbreed	5 (17.2)	24 (82.8)	1		0.58
	Known crossbreed	9 (28.1)	23 (71.9)	0.53	0.16-1.83	0.32
	Breed	47 (21.9)	168 (78.1)	0.74	0.27-2.06	0.57
	Missing	0	0			
**Breed type (UK Kennel Club)**	Toy	12 (40.0)	18 (16.0)	1		0.06
	Crossbreed	14 (23.0)	47 (77.1)	2.24	0.87-5.75	0.09
	Gundog	8 (11.9)	59 (88.1)	4.92	1.74-13.89	0.003
	Hound	3 (37.5)	5 (62.5)	1.11	0.22-5.54	0.90
	Pastoral	9 (25.0)	27 (75.0)	2.00	0.70-5.72	0.20
	Terrier	3 (12.5)	21 (87.5)	4.67	1.14-19.17	0.03
	Unrecognised	9 (31.0)	20 (69.0)	1.48	0.51-4.33	0.47
	Utility	2 (25.0)	6 (75.0)	2.00	0.32-11.61	0.44
	Working	1 (7.7)	12 (92.3)	8.00	0.92-69.84	0.06
	Missing	0	0			
Size*	Toy/small	29 (30.2)	67 (69.8)	1		0.06
	Medium	18 (19.0)	77 (81.1)	1.85	0.94-3.63	0.07
	Large/giant	14 (16.7)	70 (83.3)	2.16	1.05-4.45	0.04
	Missing	0	1			
**Age (continuous)**	Years	Mean 6.95	Mean 6.60	0.98	0.91-1.05	0.54
	Missing	2	8			
**Sex**	Male	29 (21.6)	105 (78.4)	1		
	Female	32 (22.5)	110 (77.5)	0.95	0.54-1.68	0.86
	Missing	0	0			
**Neutered**	No	19 (20.9)	72 (79.1)	1		
	Yes	41 (22.8)	139 (77.2)	0.89	0.48-1.65	0.72
	Missing	1	4			
**Owned since a puppy less than 12 weeks**	No	12 (14.5)	71 (85.5)	1		
	Yes	49 (25.7)	142 (74.4)	0.49	0.25-0.98	**0.04**
	Missing	0	2			
**Dog source**	Breeder	43 (25.9)	123 (74.1)	1		
	Other	18 (16.7)	90 (83.3)	1.75	0.95-3.23	0.08
	Missing	0	2			
**Dog duties**	Shared	34 (27.6)	89 (72.4)	1		
	One main person	26 (17.1)	126 (82.9)	1.85	1.04-3.30	**0.04**
	Missing	1	0			

*Sizes were defined in the questionnaire with examples: Toy, Small (terrier), Medium (collie/spaniel) Large (Labrador/GSD), Giant (Great Dane)

**Table 2:** Univariable analysis of household demographic factors associated with daily dog walking

Variable		<1/day	1+/day	OR	95 % CI	P
		n (%)	n (%)			
**Number of dogs**	Single	30 (17.4)	142 (82.6)	1		
	Multiple	31 (29.8)	73 (70.2)	0.50	0.28-0.88	**0.02**
	Missing	0	0			
**Own a horse**	No	57 (21.4)	209 (78.6)	1		
	Yes	4 (40.0)	6 (60.0)	0.41	0.11-1.50	0.18
	Missing	0	0			
**House type**	Detached	35 (20.1)	139 (79.9)	1		
	Attached	26 (25.5)	76 (74.5)	0.74	0.41-1.31	0.30
	Missing	0	0			
**Number of people in household**	1-2	15 (11.2)	119 (88.8)	1		
	3 or more	46 (32.6)	95 (67.4)	0.26	0.14-0.49	**<0.001**
	Missing	0	1			
**Presence of adult males**	No	2 (4.9)	39 (95.1)	1		
	Yes	59 (25.7)	171 (74.4)	0.15	0.03-0.63	**0.01**
	Missing	0	5			
**Presence of adult females**	No	1 (9.1)	10 (90.9)	1		
	Yes	60 (23.1)	200 (76.9)	0.33	0.04-2.66	0.30
	Missing	0	5			
**Household age category (see** [30]**)**	1 Over 60s	10 (17.5)	47 (82.5)	1		**0.02**
	2 Families	17 (30.4)	39 (69.6)	0.49	0.20-1.19	0.11
	3 Families	21 (32.3)	44 (67.7)	0.45	0.19-1.05	0.07
	4 Singles/couples adult	4 (7.55)	49 (92.5)	2.61	0.76-8.89	0.13
	5 Young families	4 (26.7)	11 (73.3)	0.59	0.15-2.22	0.43
	6 Older families	5 (17.2)	24 (82.8)	1.02	0.31-3.33	0.97
	Missing	0	1			
**Household occupation category (see** [30]**)**	1 Sales	7 (53.9)	6 (46.1)	1		**0.01**
	2 Skilled trade	9 (30.0)	21 (70.0)	2.72	0.71-10.41	0.14
	3 Administrative and secretarial	9 (25.0)	7 (75.0)	3.50	0.93-13.18	0.06
	4 Retired	5 (9.1)	50 (90.9)	11.67	2.80-48.57	**0.001**
	5 Personal service	3 (10.3)	26 (89.7)	10.11	2.01-50.98	**0.001**
	6 Associate professional	11 (30.7)	25 (69.4)	2.65	0.72-9.74	0.14
	7 Process/plant and machines and elementary	10 (29.4)	24 (70.6)	2.80	0.75-10.45	0.13
	8 Professional	3 (13.6)	19 (86.4)	7.39	1.44-37.88	**0.02**
	9 Managers and senior officials	4 (20.0)	16 (80.0)	4.67	0.99-21.89	**0.05**
	Missing	0	1			
**Presence of person unemployed/retired/looking after family**	No	37 (22.6)	127 (77.4)	1		
	Yes	24 (21.4)	88 (78.6)	1.07	0.60-1.91	0.82

Where cells had zero cases, statistics are not presented, and where cells have only one case, presented statistics should not be considered reliable

**Table 3:** Univariable analysis of management factors associated with daily dog walking

Variable		<1/day	1+/day	OR	95 % CI	P
		n (%)	n (%)			
**Dog access when people in the house**	Everywhere	32 (20.7)	123 (79.4)			
	Everywhere except bedroom	6 (22.2)	21 (77.8)			
	Downstairs only	13 (23.6)	42 (76.4)			
	Kitchen only	3 (27.3)	8 (72.7)			
	Living area only	2 (28.6)	5 (71.4)			
	Utility room only	0 (0)	1 (100)			
	Outside only	0 (0)	5 (100)			
	Other	5 (35.7)	9 (64.3)			
	Missing	0	1			
**Dog access when people in the house recoded**	Unrestricted or mild restriction	51 (21.2)	189 (78.8)	1		
	Restriction to one or a few places	10 (28.6)	25 (71.4)	0.67	0.30-1.50	0.33
	Missing	0	1			
**Dog lies on furniture**	Never/rarely	33 (26.0)	94 (74.0)	1		
	Sometimes/often	27 (19.9)	109 (80.2)	1.42	0.79-2.53	0.24
	Missing	1	12			
**Dog lies on laps**	Never/rarely	23 (16.0)	121 (84.0)	1		
	Sometimes/often	35 (29.7)	83 (70.3)	0.45	0.25-0.82	**0.01**
	Missing	3	11			
Amount of interaction* with people per day	Up to 1 h	13 (23.6)	42 (76.4)	1		0.82
	1-2 h	14 (19.7)	57 (80.3)	1.26	0.54-2.96	0.60
	2-4 h	12 (19.4)	50 (80.7)	1.29	0.53-3.13	0.57
	Over 4 h	21 (24.7)	64 (75.3)	0.94	0.43-2.09	0.87
	Missing	1	2			
**Play with dog in garden**	Never/rarely	0 (0)	11 (100)			
	Sometimes/often	61 (23.2)	202 (76.8)			
	Missing	0	2			
**Attended training classes**	Never	57 (22.3)	199 (77.7)			
	Ever	0 (0)	8 (100)			
	Missing	4	8			
**Dog been to vet in past year**	No	16 (35.6)	29 (64.4)	1		
	Yes	45 (19.7)	183 (80.3)	2.24	1.12-4.48	**0.02**
	Missing	0	3			
**Dog vaccinated in past year**	No	29 (27.9)	75 (72.1)	1		
	Yes	32 (18.9)	137 (81.1)	1.66	0.93-2.94	0.09
	Missing	0	3			
**Dog had check up in past year**	No	49 (25.0)	147 (75.0)	1		
	Yes	12 (15.6)	65 (84.4)	1.81	0.90-3.62	0.10
	Missing	0	3			
**Dog seen vet for health problem related to walking in past year**	No	58 (23.6)	188 (76.4)	1		
	Yes	3 (11.1)	24 (88.9)	2.47	0.72-8.49	0.15
	Missing	0	3			
**Dog flea treatment last 3 months**	No	28 (22.4)	97 (77.6)	1		
	Yes	30 (21.1)	112 (78.9)	1.08	0.60-1.93	0.80
	Missing	3	6			
**Dog worm treatment last 3 months**	No	25 (22.9)	84 (77.1)	1		
	Yes	33 (21.0)	124 (79.0	1.12	0.62-2.02	0.71
	Missing	3	7			

Where cells had zero cases, statistics are not presented, and where cells have only one case, presented statistics should not be considered reliable

*Interaction defined as (e.g. games, cuddles, training, grooming, not just resting in the same room)

**Table 4:** Univariable analysis of reasons for getting dog* associated with daily dog walking

Variable		<1/day	1+/day	OR	95 % CI	P
		n (%)	n (%)			
**Companionship**	No	21 (25.6)	61 (74.4)	1		
	Yes	40 (20.6)	154 (79.4)	1.33	0.72-2.43	0.36
	Missing	0	0			
**Protection**	No	54 (24.1)	170 (75.9)	1		
	Yes	7 (13.5)	45 (86.5)	2.04	0.87-4.79	0.10
	Missing	0	0			
**Hobby**	No	58 (25.3)	171 (74.6)	1		
	Yes	3 (6.4)	44 (93.6)	4.97	1.49-16.63	**0.01**
	Missing	0	0			
**Showing or breeding**	No	61 (22.7)	208 (77.3)			
	Yes	0 (0)	7 (100)			
	Missing	0	0			
**Exercise**	No	44 (25.0)	132 (75.0)	1		
	Yes	17 (17.0)	83 (83.0)	1.63	0.87-3.04	0.13
	Missing	0	0			
**Working dog**	No	61 (22.9)	205 (77.1)			
	Yes	0 (0)	10 (100)			
	Missing	0	0			
**Always had a dog**	No	35 (22.6)	120 (77.4)	1		
	Yes	26 (21.5)	95 (78.5)	1.07	0.60-1.89	0.83
	Missing	0	0			
**Family member wanted dog**	No	38 (19.3)	159 (80.7)	1		
	Yes	23 (29.1)	56 (70.9)	0.58	0.32-1.06	0.08
	Missing	0	0			
**Gift**	No	60 (22.2)	210 (77.8)	1		
	Yes	1 (16.7)	5 (83.3)	1.43	0.16-12.46	0.75
	Missing	0	0			

Where cells had zero cases, statistics are not presented, and where cells have only one case, presented statistics should not be considered reliable

*Multiple reasons could be indicated

**Table 5:** Univariable analysis of dog behaviour factors associated with daily dog walking

Variable		<1/day	1+/day	OR	95 % CI	P
		n (%)	n (%)			
**Barks at visitors**	Never/rarely	21 (21.9)	75 (78.1)	1		
	Sometimes/often	37 (22.8)	125 (77.2)	0.95	0.52-1.74	0.86
	Missing	3	15			
**Growls at visitors**	Never/rarely	48 (22.0)	170 (78.0)	1		
	Sometimes/often	6 (22.2)	21 (77.8)	0.99	0.38-2.59	0.98
	Missing	7	24			
**Growls at household members**	Never/rarely	47 (20.7)	180 (79.3)	1		
	Sometimes/often	8 (53.3)	7 (46.7)	0.23	0.08-0.66	**0.01**
	Missing	6	28			
**How likely to greet person**	Never/rarely	15 (23.4)	49 (76.6)	1		
	Sometimes/often	45 (21.6)	163 (78.4)	1.11	0.57-2.16	0.76
	Missing	1	3			
**How likely to greet dog**	Never/rarely	17 (27.0)	46 (73.0)	1		
	Sometimes/often	43 (20.8)	164 (79.2)	1.41	0.74-2.70	0.30
	Missing	1	5			
**Playful with dogs**	Never/rarely	23 (27.1)	62 (72.9)	1		
	Sometimes/often	34 (20.7)	130 (79.3)	1.42	0.77-2.61	0.26
	Missing	4	23			
**Aggressive to dogs**	Never/rarely	41 (22.5)	141 (77.5)	1		
	Sometimes/often	14 (20.9)	53 (79.1)	1.10	0.56-2.18	0.78
	Missing	6	21			
**Eats raw carcasses on a walk**	Never	54 (23.6)	175 (76.4)	1		
	Rarely/sometimes/often	7 (15.6)	38 (84.4)	1.68	0.71-3.97	0.24
	Missing	0	2			
**Rolls in carcasses/faeces on a walk**	Never/rarely	48 (23.5)	156 (76.5)	1		
	Sometimes/often	13 (19.1)	55 (80.9)	1.30	0.66-2.58	0.45
	Missing	0	4			
**Play fetch games**	Never/rarely	12 (25.5)	35 (74.5)	1		
	Sometimes/often	44 (20.6)	170 (79.4)	1.32	0.64-2.76	0.45
	Missing	5	10			
**Play tug-of-war games**	Never/rarely	16 (20.0)	64 (80.0)	1		
	Sometimes/often	41 (23.2)	136 (76.8)	0.83	0.43-1.59	0.57
	Missing	4	15			
**Play hide-and-seek games**	Never/rarely	36 (22.6)	123 (77.4)	1		
	Sometimes/often	16 (20.3)	63 (79.8)	1.15	0.59-2.24	0.68
	Missing	9	29			
**Play rough-and-tumble games**	Never/rarely	20 (19.8)	81 (80.2)	1		
	Sometimes/often	37 (24.0)	117 (46.0)	0.78	0.42-1.44	0.43
	Missing	4	17			
**Play chase games**	Never/rarely	15 (14.9)	86 (85.2)	1		
	Sometimes/often	41 (28.1)	105 (71.9)	0.45	0.23-0.86	**0.02**
	Missing	5	24

**Table 6:** Univariable analysis of walking behaviour factors associated with daily dog walking

Variable		<1/day	1+/day	OR	95 % CI	P
		n (%)	n (%)			
**Roaming**	Securely confined	46 (20.4)	180 (79.7)	1		
	Has escaped or allowed to roam	15 (32.3)	33 (68.8)	0.56	0.28-1.12	0.10
	Missing	0	2			
**Dog ever on a lead**	No	1 (6.3)	15 (93.8)	1		
	Yes	59 (22.8)	200 (77.2)	0.23	0.03-1.75	0.15
	Missing	1	0			
**Lead type used**	Short	35 (23.2)	116 (76.8)	1		0.98
	Extendable	15 (22.4)	52 (77.6)	1.05	0.53-2.08	0.90
	Both	9 (22.0)	32 (78.1)	1.07	0.47-2.46	0.87
	Missing	2	15			
**Dog ever allowed off lead**	No	9 (24.3)	28 (75.7)	1		0.80
	In certain areas	43 (24.2)	135 (75.8)	1.01	0.44-2.30	0.98
	Most of the time	9 (19.6)	37 (80.4)	1.32	0.46-3.76	0.60
	Missing	0	15			
**Usual walk length**	Up to 15 mins	3 (18.8)	13 (81.3)	1		0.12
	16-30 mins	16 (15.8)	85 (84.2)	1.23	0.31-4.80	0.77
	31mins- 1 h	26 (22.0)	92 (78.0)	0.82	0.22-3.08	0.77
	Over 1 h	12 (35.3)	33 (64.7)	0.42	0.10-1.78	0.24
	Missing	4	3			
Walk streets*	No	32 (24.1)	101 (75.9)	1		
	Yes	28 (19.7)	114 (80.3)	1.29	0.73-2.29	0.38
	Missing	1	0			
Walk park*	No	43 (23.6)	139 (76.4)	1		
	Yes	17 (18.3)	76 (81.7)	1.38	0.74-2.59	0.31
	Missing	1	0			
Walk beach/marsh*	No	15 (16.7)	75 (83.3)	1		
	Yes	45 (24.3)	140 (75.7)	0.62	0.33-1.19	0.15
	Missing	1	0			
Walk countryside*	No	14 (21.9)	50 (78.13)	1		
	Yes	46 (21.8)	165 (78.2)	1.00	0.51-1.98	0.99
	Missing	1	0			
Walk farmland*	No	56 (23.6)	181 (76.4)	1		
	Yes	4 (10.5)	34 (89.5)	2.63	0.89-7.73	0.08
	Missing	1	0			
**Walk regularly in same place**	No	28 (32.2)	59 (67.8)	1		
	Yes	32 (17.6)	150 (82.4)	2.22	1.23-4.01	**0.01**
	Missing	1	6			
**Walk in a group**	Never/rarely	47 (29.9)	110 (70.1)	1		
	Sometimes/often/everyday	14 (11.8)	150 (88.2)	3.20	1.67-6.16	**<0.001**
	Missing	0	0			
**Notice same people and dogs on a walk**	Never/rarely	10 (52.6)	9 (47.4)	1		
	Sometimes/often/everyday	50 (19.6)	205 (80.4)	4.56	1.76-11.80	**0.002**
	Missing	1	1			
**Pick up score** ^**a**^	Continuous	Median 16	Median 15	0.89	0.76-1.05	0.17
	Missing	13	24			
**Pick up categorised**	Never/rarely	1 (20.0)	4 (80.0)	1		0.83
	Varies by location	21 (18.4)	93 (81.6)	1.11	0.12-10.42	0.93
	Always everywhere	26 (21.7)	94 (78.3)	0.90	0.10-8.44	0.93
	Missing	13	24			

Where cells had zero cases, statistics are not presented, and where cells have only one case, presented statistics should not be considered reliable

*Multiple typical walking areas could be indicated

^a^Calculated from score 0–4 (Never, rarely, sometimes, often, always) in 4 contexts (street, public path, park, countryside)

Variables *P* < 0.3 on univariable analysis and with sufficient data (no cells with 0 or 1) were used for multivariable model building using backwards elimination. This was first conducted in smaller models of grouped factors as presented in the tables, then combined and further reduced. Variables remained in the model if they were significant (*P* < 0.05) or if removal/addition resulted in substantial change to the effect of other variables. The fit of the final model was assessed using the Hosmer-Lemeshow statistic and classification of percentage correctly predicted by the model.

When building the multivariable models, a number of decisions were made for practical purposes. The walking variables ‘walk regularly in same place’, ‘walk in a group’, and ‘notice same people and dogs on a walk’ were not used as it was deemed likely that these strong associations were, at least in part, due to reverse causality (as a result of walking regularly). Due to small groups and wide confidence intervals the ‘household occupation category’ variable was also excluded from the final model building process. Furthermore, the decision was made to use ‘size’ of dog instead of ‘breed type (UK Kennel Club)’ and ‘owned since a puppy’ instead of ‘dog source’ as these were collinear and better described in terms of the context of the outcome, by the former variables.

Finally, due to the nature of a minority of the dogs in the dataset being non-independent as they lived together in households (62 % single dog, 32 % two dogs, 5 % three dogs), we re-ran the model randomly selecting only one dog from each multi-dog household; the results and conclusions drawn were largely and qualitatively consistent with the model containing all dogs so only those findings are presented here.

## Results

### Walk frequency

One dog (0.4 %) was reportedly never walked, 6 (2.2 %) less than once a week, 8 (2.9 %) once a week, 46 (16.6 %) several times a week, 82 (29.5 %) once a day, 90 (32.4 %) twice a day, 37 (13.3 %) three times a day and 8 (2.9 %) ‘other’; of these, 6 reported walking 4 times a day or more, one was an unvaccinated puppy that was not walked so treated as missing data, and for one it was not possible to estimate usual walk frequency from the answer given. For one dog this question was not answered. Thus, of 276/279 dogs with clear data provided, 61 (22.1 %) were reported to be walked less than once a day, and 215 (77.9 %) at least once a day or more.

An alternative to walking a dog may be letting it roam without the owner: the majority of dogs (228; 82.6 %) were reported to be confined to a secure area; 34 (12.3 %) generally confined but have escaped in the past, 11 (4.0 %) not confined but generally choose not to roam; and 3 (1.1 %) allowed to roam freely. The majority of owners (157; 70.7 %) also reported that household members interact or play with the dog in the garden area ‘often’, which could be considered an alternative form of physical activity with the dog.

### Walk length

Households reported that dog walks were, on ‘average’, 16-30mins (88; 40.6 %) or 31-60mins (91; 41.9 %) in length; it was rarer for walk lengths to be only up to 15mins (14; 6.5 %) or over 1 h (24; 11.1 %).

### Place of dog walking

The most popular places for walking dogs were in the countryside (165; 74.7 % of households indicated), on the beach (141; 63.8 %) or on the streets (119; 53.9 %). In contrast, parks (81; 36.7 %) and farmland (31; 14.0 %) were less popular. It was very common for households to report walking regularly (mostly daily) in the same place (150; 69.4 %). Travelling in the car or public transport to other areas to dog walk was also common; 86 households (39.5 %) did this several times a month or more but 60 (27.5 %) never.

### Off/On-leash while on walks

Sixteen (5.8 %) dogs were never walked on a leash. Short leashes were most commonly used (151; 58.1 %) with 67 (25.8 %) being walked on an extendable flexi-leash and 42 (16.2 %) a mixture of both. The majority of dogs were allowed to walk off-leash in certain areas (178; 67.9 %) or most of the time (46; 17.6 %) with only 38 dogs (14.5 %) being kept on a leash all of the time.

### Dog behaviour on walks

The majority of dogs were reported to ‘often’ (133; 48.5 %) greet and make physical contact when they see a person; 17 (6.2 %) were reported to do this ‘never’ or 47 (17.2 %) ‘rarely’. If they were to see another dog, dogs were reported to ‘often’ (106; 39.0 %) or ‘sometimes’ (101; 37.1 %) greet them and make physical contact; 23 (8.5 %) were reported to ‘never’ or 42 (15.4 %) ‘rarely’ do this. On a walk, 164 dogs (59.0 %) were reported to ‘often’ or ‘sometimes’ play with dogs and 68 (24.5 %) ‘often’ or ‘sometimes’ be aggressive to dogs. Whilst the former may be considered an enjoyable part of the dog walking experience for the owner and dog, the latter behaviour may be considered undesired by and thus a disincentive to talking the dog for a walk. Further potentially undesirable behaviours were also investigated; however, the majority of dogs were reported to never find and eat raw carcasses (231; 83.7 %), roll in faeces or carcasses (136; 49.6 %) or eat dog faeces (246; 89.5 %).

### Reasons for getting a dog

For 82 dogs (36.4 %) the reported reason the owner chose to get a dog was for exercise; the most common reason indicated was companionship (154;68.4 %).

### Dog walking and social interactions

Twenty seven (12.2 %) dogs were walked with other dogs known to them either ‘everyday’ or ‘often’, whereas 85 (38.3 %) never did this. However, 138 (62.7 %) reported seeing the same people and their dogs (otherwise unknown to them) on dog walks ‘everyday’ or ‘often’.

### Univariable analysis

Univariable associations between factors and dog being walked once a day or more are reported in Tables 1, 2, 3, 4, 5 and 6. Statistical findings are not reported when no dogs are reported in a category. Cells where only one dog is reported were considered unreliable and not taken forward from multivariable analysis, although calculated statistics are reported.

It is interesting to note that dogs that were kept outside or in a utility room, were all walked every day (Table 3). However when this variable ‘Dog access when people in the house’ was regrouped to ‘Unrestricted or mild restriction’ versus ‘Restriction to one or a few places’, there was no association.

Further, dogs that had ever been to training classes, were acquired to show or breed, were working dogs, and that were never/rarely played with in the garden, were all walked at least once a day (see Tables 3 and 4). Due to the lack of data for statistical analyses, these variables could not be modelled further. However for univariable analysis purposes we randomly selected one dog within each variable to change to not walked daily but the findings did not approach significance (Chi-squared P > 0.3). In addition, all dogs whose owners reported never picking up after their dog on a public path, park or street, were walked every day (data not shown).

There was strong evidence of a positive association between walking once a day or more and reporting regularly walking in a group (OR = 3.20, 95%CI 1.67-6.16, P < 0.001), or seeing recognisable dogs and owners (OR = 4.56, 95%CI = 1.76-11.80, *P* = 0.002).

### Multivariable analysis

In the final multivariable model (Table 7) daily dog walking was independently associated with: number of dogs (multiple negative); size (medium and possibly large dogs positive); number of people in the household (negative); having got the dog as a hobby (positive); dog lying on furniture (positive); dog lying on laps (negative); growling at household members (negative); and playing chase games with dog (negative).

**Table 7:** Multivariable binary logistic regression model of factors associated with daily dog walking

Variable		OR	95 % CI	P
**Number of dogs**	Single	1		
	Multiple	0.20	0.09-0.46	**<0.001**
**Number of people in household**	1-2	1		
	3 or more	0.30	0.12-0.74	**0.02**
**Reason got dog - hobby**	No	1		
	Yes	5.15	1.11-23.83	**0.04**
Size* of dog	Toy/small	1		0.10
	Medium	2.96	1.08-8.11	**0.04**
	Large/giant	2.17	0.72-6.56	0.17
**Dog lies on furniture**	Never/rarely	1		
	Sometimes/often	2.39	1.00-5.70	**0.05**
**Dog lies on laps**	Never/rarely	1		
	Sometimes/often	0.41	0.17-1.05	**0.06**
**Dog growls at household members**	Never/rarely	1		
	Sometimes/often	0.29	0.07-1.24	0.10
**Plays chase games**	Never/rarely	1		
	Sometimes/often	0.36	0.15-0.86	**0.02**

n = 212**.** Hosmer-Lemeshow =0.34. Predicted correct in classification table 81.6 %

*Sizes were defined in the questionnaire with examples: Toy, Small (terrier), Medium (collie/spaniel) Large (e.g. Labrador/GSD), Giant (Great Dane)

## Discussion

**Fig. 1:**
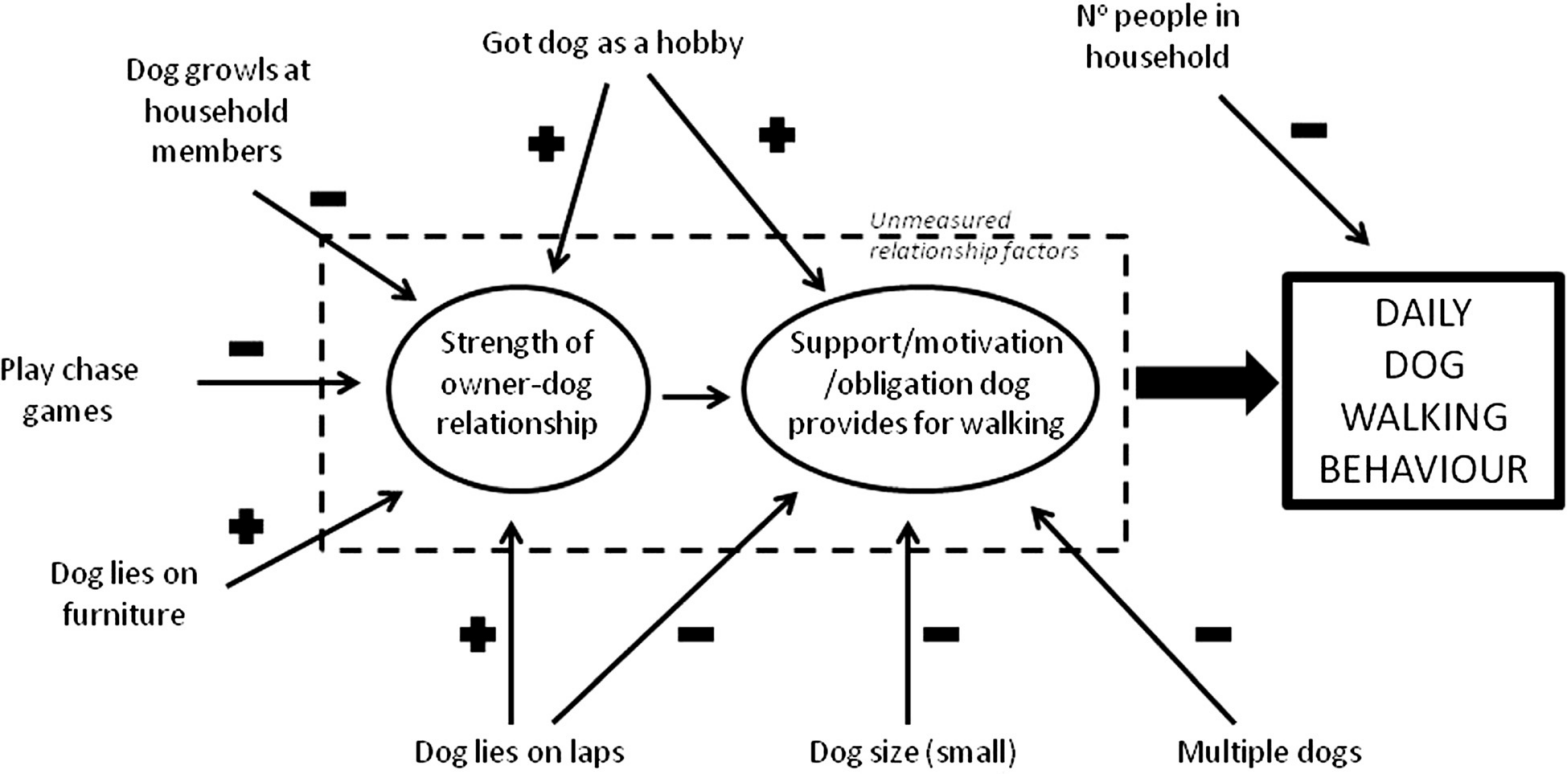
Conceptual map of behavioural and demographic variable influences on daily dog walking via the dog-human relationship and support/motivation/obligation provided by the dog for walking

This study is the first to describe in detail dog walking behaviour in a UK dog population and has identified a number of factors independently associated with daily dog walking. Owning multiple dogs, a small dog, and increasing numbers of people in the household were negatively associated with daily dog walking, and thus may be barriers or disincentives to dog walking. Other factors associated with daily dog walking were related to the strength of the dog-human relationship, for example: acquiring the dog for the purposes of a hobby; letting the dog lie on furniture (which may indicate a closer relationship); and letting the dog lie on laps (that may reflect a relationship based more on tactile interactions and comfort than enjoying shared outdoor activities). Finally, growling at household members and playing chase were negatively associated with daily dog walking. A conceptual model of how these factors may be influencing dog walking via the relationship is presented in Fig. 1.

It is plausible that aggression might be caused by reduced exercise, but only aggression specific to household members, not other dogs or people, was associated with lower odds of walking daily; thus the association with aggression to household members is more likely to be due to problematic behaviour that can weaken the dog-human bond. Playing chase games may be a substitute activity for dog walking; however it is interesting that only chase and no other reported types of games was associated with not walking daily. This leads us to believe that there may be something in particular about chase games that is associated with a weaker human-animal bond. This may be explained by the personal observation that when working with dogs and their owners, those that ‘play chase’ are often dogs that are preferring to not go back on their leads at the end of the walk, or like to steal items and get their owners to chase them for it.

Owning multiple dogs has also been found to be a barrier to regular walking in some studies [20, 32] but not in others [14, 15, 21, 27]. Plausibly multiple dogs might be harder to manage on a walk or there may be less incentive as they have each other to play with. Our findings are also in agreement with other studies which have showed that smaller dogs are less likely to be walked than larger dogs [18]. Our study is in agreement with others showing that dog sex or neuter status is not associated with dog walking behaviour [5] but did not find any evidence of a negative association between dog age and dog walking, [24, 33, 34].

Furthermore our findings agree that there is no evidence of an association between owner gender and dog walking behaviour [5]. However, we found evidence that having more people in the household was a barrier to daily dog walking and this may be a reflection of there being children in the household; (although our household age categories variable which included specified families with children was non-significant when building the final model and thus removed, the number of people variable may have been accounting for this). Previous literature investigating whether having dependents or other people living in the home is associated with dog walking behaviour was inconclusive overall [5].

In contrast to the expectation that getting a dog for the purpose of exercise may be associated with dog walking, of which we found no evidence, getting the dog for the purposes of a hobby does appear to be associated with daily dog walking. This is a novel finding and may indicate greater commitment to spending time with the dog. The walking variables ‘walk regularly in same place’, ‘walk in a group’, and ‘notice same people and dogs on a walk’ were not used within the model building as it was deemed likely that these strong reported associations were, at least in part, due to reverse causality (as a result of walking regularly). However this context deserves future investigation as motivation to walk may be related to the social contact provided through dog walking.

Our study lends strong support to the suggestion that the human-dog relationship is key to incentivising dog walking behaviour [5]; dog management factors such as letting the dog lie on the sofa, lie on laps and the personal factor of having acquired the dog for the purpose of a hobby, were associated with walk frequency. Due to the multivariable analysis model including size, we know that these associations are not simply due to the effect of size, eg small dogs being more likely to lie on laps. It also demonstrated that behaviour such as aggression or reported frequent chase games (which may not be conducive to a dog being perceived as obedient) are barriers to walking. Interestingly, aggression towards household members was associated with reduced walking, but not aggression towards other dogs or visitors to the household. This suggests that aggression towards the owner may be less manageable than aggression to other people/dogs when it comes to walking, and also, and most importantly, that it is likely more damaging to the human-animal bond, supporting the hypothesis of the role that the relationship plays in motivating an owner to want to walk their dog regularly. Our findings are in contrast to previous studies which found that behavioural issues are not associated with dog walking, but this was once support and motivation provided by the dog for walking are included in models [5].

It is possible that certain dog behaviours and management factors may contribute to the support and motivation for walking a dog can provide. This may also be true of demographic factors such as dog size, hence why they also do not appear important correlates when support and motivation provided by the dog for walking is accounted for in some previous studies (eg [21]). Further investigation is now required, particularly into aspects of the dog-human relationship or dog-related factors that may contribute to the feelings of support and motivation provided by a dog that can encourage dog walking behaviour. Future studies should also investigate the context of dog walk frequency for dogs that are kept outside, go to training classes, were acquired to show or breed, are working dogs, and whose owners do not pick up their dog’s faeces.

The main strength of our study is the detailed data collected allowing in-depth exploration of many aspects of dog and owner demographics, and dog and owner behaviours and management factors, compared to many previous studies. In particular, much detail was collected on dog walking practices and on dog and owner behaviours that may contribute to, or be proxies for, the strength of the dog-owner relationship. Thus new aspects relating to dog ownership and the dog-human relationship could be explored. A further strength is the use of multivariable modelling techniques to adjust for the effects of other variables and test for independence of associations, something that has not always been done in previous studies of this nature [5].

This study was limited by its relatively small sample size meaning that we were unable to investigate further some household demographics such as age and occupation, any effects of dog breed, and other management factors that did not contain enough data for further statistical analysis. In addition, the data was collected in one specific area of the UK, a semi-rural area where dogs may be walked more frequently, and thus findings may not be completely generalisable, particularly as there are cultural differences in the way dogs are owned and managed. It was also collected in the summer-autumn period, which may affect dog walking practices reported if seasonality has an influence.

This study also did not ask specific questions concerning the human-dog relationship, nor this specifically in relation to dog walking; for example support and motivation provided by the dog for walking [21]; a sense of ‘obligation’ [10]; and feelings that the dog ‘enjoys’ walking [21]. In addition, although this study measured some aspects of problem behaviour, it did not ask questions specifically pertaining to problem behaviour during walking, such as pulling on the lead (however previous evidence suggests that behavioural barriers are unimportant once the support/motivation factors are accounted for [5]). The health of the owner was also not investigated specifically. Future studies should investigate these contexts further. Working status and occupation was also difficult to investigate within this dataset. Future studies may wish to include a specific measure of whether someone in the household is often at home during the day, either due to non-employment, study, part-time work, or working from home, as this could influence dog walking strategies.

## Conclusions

This study identified that a number of factors related to the strength and nature of the owner-dog relationship are associated with daily dog walking. Future studies need to investigate further how dog demographic and behavioural factors, plus owner behavioural factors and perceptions of the dog, influence the dog-human relationship in respect to the perceived support and motivation a dog can provide for walking. This information can then lead to the design of effective interventions to promote dog walking behaviour through this relationship, and improve the health of both people and their pets.
